# Recent advances in deep learning for leukemia diagnosis: a scoping review of diagnostic modalities and fusion-based approaches

**DOI:** 10.3389/fdgth.2026.1874695

**Published:** 2026-08-07

**Authors:** P. Afsar, T. M. Navamani

**Affiliations:** School of Computer Science and Engineering, Vellore Institute of Technology (VIT), Vellore, Tamil Nadu, India

**Keywords:** deep learning, explainable AI techniques, genomic data analysis, leukemia diagnosis, medical image-based analysis, multimodal AI frameworks

## Abstract

The diagnosis of leukemia is inherently a multimodal process that combines morphological evaluation, immunophenotyping, molecular profiling and standard laboratory tests. Deep learning techniques have been adopted across all components of the diagnostic process over the past few years, particularly in microscopic image analysis. However, current research and review articles are highly fragmented, and most methods are limited to the single data modalities and benchmark-based performance analysis, which provide a limited understanding of model generalizations, clinical and real-world applicability. This scoping review presents a detailed analysis of recent studies of leukemia detection and diagnosis based on deep learning, in a structured and clinically grounded approach, and summarizes the literature on major diagnostic modalities, such as peripheral blood and bone marrow image analysis, gene expression and multi-omics modelling, flow cytometry-based learning, routine laboratory data analysis, and multimodal diagnostic approaches. However, only a limited number of studies have implemented true multimodal integration. Instead of just evaluating reported accuracy, this scoping review critically analyzes the impact of dataset design, modality-related bias, and validation methods, and model interpretability on the reliability and translational capabilities of the suggested approaches. New methodological developments, including transformer-based architectures, attention-based multi-instance learning, foundation models and explainable artificial intelligence, are presented in connection to robust, cross-dataset generalization and clinical reliability. Combining evidence representing traditionally separated lines of research, this scoping review highlights the research gaps in the methodology and the translation of AI methods to the real world, for the use of AI in leukemia. Moreover, it provides a clear roadmap for research in this area, emphasising modality-aware fusion, external validation, regulatory-aware, and human-in-the-loop decision support.

## 1 Introduction

Leukemia is a heterogeneous group of hematological malignancies that affects the blood and bone marrow. The current diagnostic practice is based on an integrated approach to morphology, immunophenotyping, cytogenetics, and molecular genetics based on the guidelines of the World Health Organization (WHO) Classification of Hematolymphoid Tumors (5th Edition) and International Consensus Classification (ICC) (1). Notably, these frameworks define genetically determined entities of leukemia and, in some cases, do not require a strict blast percentage cut-off in the presence of certain molecular abnormalities, a shift towards genomics-based taxonomy. No single modality is adequate in the routine hematopathology, a diagnosis is formed in a multimodal synthesis that includes the microscopic analysis of bone marrow or peripheral blood smears, flow cytometric immunophenotyping, standard cytogenetics and, increasingly, next-generation sequencing-based molecular profiling. This multimodal character is inherent and highlights the complexity and clinical significance of proper diagnosis of leukemia. This multimodal diagnostic paradigm also reflects the new direction of artificial intelligence research, in which computational models are increasingly combining heterogeneous sources of biomedical data to better capture the complexity of disease.

Despite these advances in technology, conventional diagnostic processes remain labour-intensive and rely on expert interpretation. Morphological evaluation is also prone to inter-observer variation, especially in the differentiation of morphologically similar blast populations and dysplastic appearances (2). Although molecular and cytogenetic tests are very informative and are the main focus of modern classification systems of leukemia, they are resource-intensive and not available everywhere, particularly in low-resource environments (3). Moreover, modern risk stratification models, including the European LeukemiaNet (ELN) 2022 guidelines, incorporate molecular changes into treatment decision-making, underscoring the need for rapid and accurate genomic interpretation. Such practical limitations have inspired the creation of Artificial Intelligence (AI) based decision-support systems to enhance the speed, consistency, and scalability of diagnostics (4).

Over the past decade, Deep Learning (DL) has achieved impressive success in the automation of the image-based aspects of the leukemia diagnostic pipeline (5). Convolutional Neural Network (CNN) models have achieved high performance in peripheral blood smear classification tasks on benchmark datasets, including ALL-IDB and C-NMC 2019.

Studies utilizing curated cell-level datasets, including ALL-IDB2, have reported near-perfect binary classification accuracy for Acute Lymphoblastic Leukemia (ALL) detection in controlled experimental settings (6). Similarly, deep learning frameworks incorporating YOLO and EfficientNet-based frameworks have been used for the automated detection and subtype differentiation of Acute Myeloid Leukemia (AML) from peripheral blood and bone marrow images. Recently, large-scale Whole-Slide Image (WSI) studies have demonstrated the feasibility of predicting therapy-relevant genetic alterations directly from morphology (7, 8), whereas annotation-free mutation prediction using multiple-instance learning has also been reported (3, 5). These advances suggest that morphological deep learning models are beginning to bridge phenotypic and genotypic representations in controlled research settings.

Apart from advances in imaging-based methods, machine learning and deep learning approaches have been widely used for transcriptomic and multi-omic classification and prognosis in leukemia. Large-scale RNA-sequencing-based subtype classifiers have shown strong multiclass classification performance in large transcriptomic datasets (9). LASSO-Cox and ensemble survival learning models using The Cancer Genome Atlas (TCGA) and Gene Expression Omnibus (GEO) data have been developed to predict survival and treatment response in Acute Myeloid Leukemia (AML) (10, 11). Multi-omic clustering methods have also demonstrated the power of integrating transcriptomic, methylation, mutation, and copy-number information to define molecular subtypes, in addition to the existing ELN 2022 criteria (12). These molecular AI models often have good prognostic discrimination, with Area Under the Curve (AUC) values above 0.85 in cross-cohort validations.

Beyond the use of artificial intelligence for imaging and modeling gene expression, AI has also been utilized to develop models for the automated detection of types of leukemia and the prediction of molecular aberrations from flow cytometry data, without the need for manual gating. These methods further integrate AI into routine immunophenotyping and demonstrate the growing use of computational techniques in several diagnostic techniques (13).

In addition to the use of imaging and omics data, machine learning models that use clinical and hematology parameters have also shown promise in classifying leukemia subtypes, further indicating the variety of AI systems that can be utilized in the care of leukemia patients (4).

Despite these technological advances, the existing literature remains scattered. Many imaging studies are published using small and heavily oversampled public benchmarks with limited external validation, potentially limiting the generalizability to other real-world domains and imaging centers (14). On the other hand, the design of transcriptomic or multi-omic models often relies heavily on retrospective public datasets, such as The Cancer Genome Atlas (TCGA) or BeatAML, with limited prospects and availability for real-world diagnostic applications (15). Furthermore, most existing reviews focus on metrics reported to assess models, such as accuracy, sensitivity and the area under the curve (AUC), rather than issues of dataset bias, robustness across multiple centers, interpretability, regulatory, and clinical operational considerations. This gap results in a translation gap for the implementation of superior algorithms. More importantly, existing leukemia Artificial Intelligence (AI) studies have been largely isolated from each other in terms of modality, with an emphasis on imaging or molecular models, rather than diagnostic systems. This approach may lead to benchmarks being fine-tuned rather than truly representing integrated diagnostic processes typical of modern hematopathology lab standards.

As the diagnosis of leukemia is inherently multimodal, the development of unimodal AI solutions is likely insufficient to model the complexity of real-world diagnosis. Recent multimodal approaches that combine imaging, transcriptomic and clinical information have started to show the promise of modality-aware decision support. However, there is no scoping review of these approaches from a clinically relevant perspective.

Although there are multiple review articles that have discussed the applications of AI in the context of specific diagnostic modalities for leukemia, the majority of these survey papers have discussed each of these domains in isolation. Therefore, there is a lack of review articles that discuss the relationship between each of these unimodal AI systems in relation to the integrated modality that is used for the diagnosis of leukemia.

Specifically, this scoping review (i) integrates existing knowledge from different domains in imaging, immunophenotyping, molecular, and clinical domains within a clinical framework (ii) evaluation of deep learning methods for leukemia through validation, generalizability, and interpretability studies (iii) evaluates the translational potential of current systems in the context of clinical practice, and (iv) outlines steps for developing modality-aware, explainable and clinically integrated artificial intelligence systems. By reviewing these disparate areas of research, this scoping review establishes a roadmap for future deep learning approaches to leukemia diagnosis.

## 2 Methods

This section outlines the methodology used to identify, select and synthesise the studies that examined the application of artificial intelligence in the diagnosis and prognosis of leukemia. The PRISMA-ScR guidelines (16) were followed to conduct the review in a transparent and reproducible manner. The study design, search strategy, eligibility criteria, selection process, and data synthesis methods are described in the following subsections.

### 2.1 Study design

This scoping review was conducted in accordance with the Preferred Reporting Items for Systematic Reviews and Meta-Analyses Extension for Scoping Reviews (PRISMA-ScR) guidelines. The final PRISMA-ScR checklist is included in Supplementary Material S2. This study aimed to review recent developments in Artificial Intelligence (AI)-based approaches to leukemia diagnosis and prognostic modeling in various diagnostic modalities.

### 2.2 Search strategy

A literature search was performed in PubMed, Scopus, Web of Science, and IEEE Xplore to identify studies published between January 1, 2023, and February 2026. This time frame was chosen to reflect recent developments in AI-based leukemia diagnosis, including transformer-based architectures, multimodal learning frameworks, explainable AI techniques, and related deep learning methodologies. Where relevant, earlier landmark studies and benchmark datasets were used as background references, but the literature search aimed to highlight the more recent methodological developments and research trends. The databases were chosen to complement each other with respect to biomedical, clinical, computational and engineering research relevant to leukemia diagnosis. PubMed was selected because it covers a wide range of biomedical and clinical literature. At the same time, Scopus and Web of Science were added because they index a wide range of peer-reviewed publications across many disciplines. IEEE Xplore was chosen to capture studies on artificial intelligence, machine learning and deep learning, which are often published in engineering journals. ScienceDirect was not searched separately because its content is heavily indexed in Scopus, and Google Scholar was not searched due to the lack of reproducibility in its search and systematic record retrieval. The search strategy was a combined controlled vocabulary (terms such as Leukemia, Artificial Intelligence, and Machine Learning) and free-text keywords, such as deep learning, leukemia, artificial intelligence methodologies, and multimodal diagnostic data. Optimization of retrieval was achieved using database-specific syntax and Boolean operators. Only full-length, peer-reviewed articles in English were included. Clinical trial registries and gray literature were not considered because the review was limited to published computational modeling studies. All database-specific search terms are presented in Supplementary Table S1.

### 2.3 PICOS framework and research question

The PICOS framework was used to formulate the review question to explore the use of artificial intelligence-based multimodal models in the diagnosis and prognosis of leukemia.

- Population (P):Patients diagnosed with or suspected of having leukemia.
- Intervention (I): Artificial intelligence, machine learning, and deep learning models applied to diagnose, classify, prognosis prediction, and classify risks in leukemia.
- Comparator (C): Existing AI-based approaches reported in the included studies, where applicable.
- Outcomes (O): Diagnostic performance measures such as accuracy, sensitivity, specificity, Area Under the Curve (AUC), precision, F1-score, robustness and interpretability.
- Study Design (S): Original research articles.

### 2.4 Eligibility criteria

The studies were included if they used artificial intelligence, machine learning, or deep learning techniques to diagnose, classify, predict prognosis, or stratify risk in leukemia. Eligible studies utilized imaging data, such as peripheral blood smears, bone marrow aspirates, and whole-slide images; flow cytometry data; molecular or genomic data, including gene expression, mutation profiling, and multi-omics; as well as clinical laboratory parameters. Only full-length, peer-reviewed journal articles published in English and reporting quantitative performance metrics, such as accuracy, Area Under the Curve (AUC), sensitivity, specificity, and F1-score, were included. Articles that were not specifically focused on leukemia did not employ artificial intelligence or machine learning as a central component, had purely therapeutic or biological objectives involving treatment response prediction, drug discovery, therapeutic interventions, or biological investigations without computational modeling, or were review articles, editorials, or conference abstracts were excluded. Studies were excluded if they did not provide sufficient methodological or performance information for data extraction and comparison. This included studies that lacked essential methodological details, such as the model architecture or algorithm used, the dataset source or sample size, or at least one quantitative diagnostic performance metric required by the inclusion criteria. For instance, a study that describes an AI-based leukemia classification model but does not explicitly state the source of the dataset or the number of samples, the validation protocol, the architecture of the model or algorithm, or at least one quantitative diagnostic performance metric that would be needed for comparative synthesis would be considered a borderline case. Without the ability to identify these key elements following full-text assessment, the study would be excluded, as it would not be possible to standardise data extraction and make reliable comparisons between studies. Studies for which eligibility was unclear were evaluated using the predefined inclusion and exclusion criteria, and a final screening decision was made.

### 2.5 Study selection and data extraction

All records obtained from the databases were exported and combined into a reference management system, where duplicate records were detected and removed prior to screening. The remaining records were filtered separately based on their titles and abstracts, as per the predetermined eligibility criteria. Articles considered potentially eligible were reviewed in full text to determine final inclusion. Two authors independently screened the titles, abstracts, and full-text articles according to the predefined eligibility criteria. To assess the reliability of the title and abstract screening stage, a random 20% sample of the 1,317 screened records (*n* = 264) was independently evaluated by both authors using the same predefined inclusion and exclusion criteria. Percentage agreement, calculated from the authors’ independent screening decisions before consensus resolution, was 96.21% (254 of 264 records), indicating a high level of agreement between the two authors. Disagreements during the screening and eligibility assessment process were resolved through discussion and consensus.

**Figure 1:**
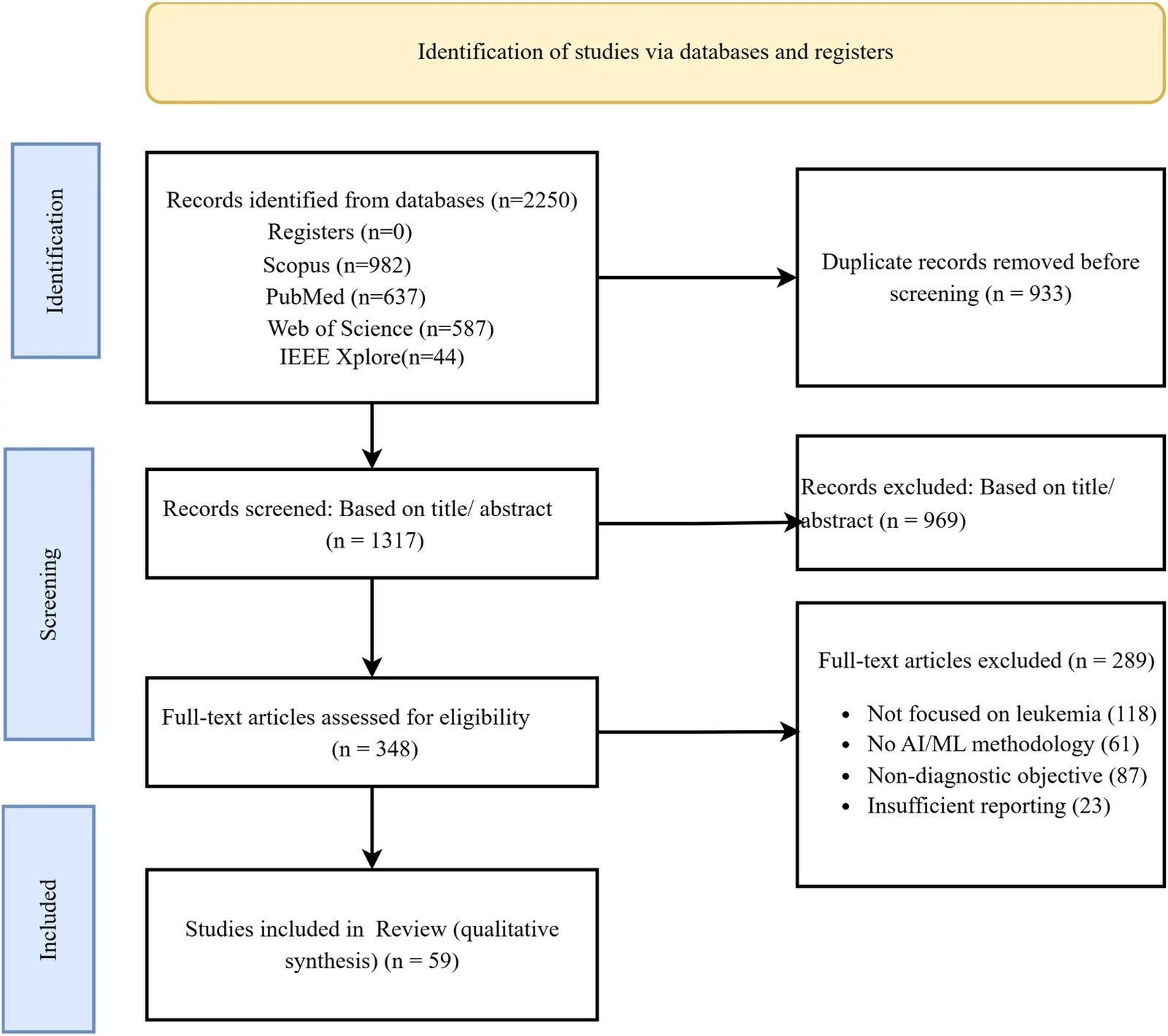
PRISMA-ScR flow diagram of the study selection process.

The pertinent information was carefully gathered on each of the included studies, such as leukemia subtype and primary task (including diagnosis, classification, or prognosis), data modality (imaging, genomic, flow cytometry, clinical laboratory, or multimodal), data source, sample size, model architecture, validation plan, and reported performance metrics. Data were extracted and analysed to determine the trends in the methods used and the validity of the validations. Figure 1 represents the selection of studies and the reasons for the exclusion of the studies at the full-text stage.

### 2.6 Data synthesis

Due to the variety of study designs, AI approaches, datasets, and outcomes, it is not possible to perform a meta-analysis of the studies. Instead, a narrative synthesis of the studies is performed. The studies were grouped by data type as image, genomic, and clinical. Within each group, studies were reviewed to determine the evolution of algorithms, the validation methods used, and the dataset sizes required. Finally, the readiness of those methods for use in clinical settings was reviewed to identify potential barriers to translating those AI approaches.

## 3 Results

We conducted a literature search and identified a series of recent studies that explored the use of artificial intelligence for leukemia diagnosis and prognosis across different biomedical data modalities. This section describes the study selection process, the key research characteristics and the emerging trends in methodology. It also examines the data modalities, datasets, and artificial intelligence models used in leukemia studies, and the growing trend toward multimodal artificial intelligence systems for multimodal diagnostic analysis.

### 3.1 Selection of eligible studies

The initial search yielded 2,250 records. We removed 933 duplicates and assessed 1,317 studies based on titles and abstracts. Then, 348 full-text articles were assessed for eligibility, and 289 were excluded based on pre-determined criteria, including no use of AI or ML methodology, no focus on leukemia, no diagnostic objectives or insufficient methodological detail. Ultimately, 59 studies were included in the qualitative synthesis. The PRISMA-ScR flow diagram (Figure 1) illustrates the study selection process. The key findings of these papers are presented in the following sections.

### 3.2 Study characteristics and research trends

The 59 studies reviewed in the study demonstrated a rapidly evolving field of research at the intersection of artificial intelligence and leukemia diagnosis in 2023–2026. The number of publications was shown to grow steadily over the years, which was accompanied by the increasing adoption of transformer-based models, foundation models and multimodal learning approaches in computational hematology. The analysis of the included studies revealed several key trends related to the clinical application of the research, the data used to train the model, and the methods used.

#### 3.2.1 Clinical focus

Most of the literature has been focused on acute leukemias. Acute Myeloid Leukemia (AML) was the most investigated, followed by Acute Lymphoblastic Leukemia (ALL), and less studies were conducted on Chronic Lymphocytic Leukemia (CLL) and Chronic Myeloid Leukemia (CML). This variability can be justified by the biological and genomic diversity of acute leukemias that offer a rich ground for predictive modeling and the availability of large public genomic databases of AML and well-established imaging protocols of ALL. On the other hand, chronic leukemias, particularly CML, are typically characterised by distinct molecular drivers and diagnostic algorithms (1), which may limit the need for complex AI-based diagnostic models. In addition to the disease subtype focus, the reviewed studies also vary significantly in the types of biomedical data employed in artificial intelligence modelling.

#### 3.2.2 Data modalities

Regarding the type of input data, imaging-based methods have prevailed in this field, mainly relying on peripheral blood smear images, bone marrow aspirates, and whole-slide histopathology. The second largest category was molecular and genomic modeling, which harnessed the use of gene expression patterns, mutation data, methylation patterns, and multi-omics integration systems to classify diseases and stratify prognosis. Models and methods based on flow cytometry that use only routine clinical laboratory parameters have been relatively limited. Multimodal integration strategies are a technically advanced yet emerging research direction that involves combining two or more distinct data sources. The computational architectures used in predictive modeling of leukemia AI research are also highly dependent on the choice of these data modalities.

#### 3.2.3 Methodological trends

In other modalities, Convolutional Neural Networks (CNNs) were still the most prevalent architecture in the imaging-based leukemia research. Recent publications since 2024, however, are more likely to consider Vision Transformers (ViTs), attention-based models, and Graph Neural Networks (GNNs) to learn long-range morphological dependencies and structural relationships among cells (17–19). These changing methodological directions towards increasing sophistication of AI solutions for the problem of leukemia and indicate the general transition towards more sophisticated deep learning architectures that can process heterogeneous biomedical data.

Recent leukemia studies have increasingly adopted Vision Transformers (ViTs), attention-based architectures, and Graph Neural Networks (GNNs), which can be explained by their capacity to process global contextual information and complex spatial relationships, which are difficult to model with traditional CNNs. Transformer-based architectures use attention mechanisms to capture long-range dependencies between image regions, whereas CNNs are very good at learning local image features. Similarly, GNNs offer a flexible representation of structural interactions between cells and tissue components. Such architectures are gaining popularity for leukemia diagnosis. A comparison of CNNs and transformer-based architectures provides several practical trade-offs in the context of leukemia diagnosis. CNNs are widely used due to their computational efficiency, performance on relatively small datasets, and ability to capture local morphological features in blood smear and bone marrow images. However, they tend to be restricted to local contextual relationships within an image due to the localized nature of convolutional operations. Transformer-based models, on the other hand, use self-attention mechanisms to capture information from spatially separated regions of the image, which makes them appealing for analysing intricate cellular patterns. This benefit, however, is accompanied by more computational demands and reliance on large training datasets. GNNs can be useful in modeling relationships between cells, genes, or other biological entities as interconnected structures, which can be valuable when the relationship is diagnostically relevant. In recent years, hybrid architectures have been increasingly studied, which integrate CNN feature extraction with transformer contextual modelling, to achieve a balance between efficiency, accuracy, and representational power.

### 3.3 Data landscape and structural gaps in leukemia AI

The promise of Artificial Intelligence (AI) in the diagnosis of leukemia is inexorably tied to the quality, variety and availability of data. In comparison to imaging AI benchmarks like ImageNet, leukemia AI research is in a fragmented and modality-isolated ecosystem with a small number of cohort and institutional silos and lacking the cross-modal integrity. These studies synthesized indicate structural data constraints as one of the main determinants of reported model performance, reproducibility, and clinical applicability.

#### 3.3.1 Representative datasets across modalities

Several publicly available datasets have facilitated the creation of machine learning and deep learning models to detect leukemia and model prognostics. These data sets differ in modality, cohort size, and level of annotation. Table 1 provides an overview of the representative datasets that are typically utilized in AI studies of leukemia and their original publications.

**Table 1:** Publicly available datasets commonly used in leukemia artificial intelligence research.

Dataset	Reference	Modality	Samples/patients	Typical AI task
ALL-IDB1	(20)	Microscopy Imaging Data	108 microscopic images	Leukemia detection
ALL-IDB2	(20)	Microscopy Imaging Data	260 cell images	Leukocyte classification
C-NMC 2019	(21)	Microscopy Imaging Data	15,114 cells/118 patients	ALL vs HEM classification
AML-Cytomorphology LMU	(22)	Microscopy Imaging Data	18,365 cells/200 subjects	AML morphology classification
CPTAC-AML	(23)	Microscopy Imaging Data	AML patient slides	AML subtype classification
TCGA-LAML	(24)	Genomic and Multi-Omics Data	200 AML patients	Prognostic modeling/genomic analysis
BeatAML	(25)	Genomic and Multi-Omics Data	562 AML patients (672 samples)	Drug response prediction
TARGET-AML	(26)	Genomic and Multi-Omics Data	∼1,000 pediatric AML patients	Genomic risk prediction

As it is summarized in Table 1, publicly available datasets on leukemia AI research are highly heterogeneous in terms of data modality, cohort size, and annotation structure. Morphological analysis of leukocytes is mainly supported by imaging datasets, including ALL-IDB, C-NMC, and AML-Cytomorphology LMU, and molecular characterization and prognostic modeling are supported by genomic resources, including TCGA-LAML, BeatAML, and TARGET-AML. Incontrast, Flow cytometry and clinical laboratory modalities, on the other hand, were underrepresented in the literature reviewed. This underrepresentation is due to the relative scarcity of standardized benchmark datasets and indicates an important gap in data availability for future research. Nevertheless, most datasets remain modality-specific, and publicly available cohorts combining imaging, genomic, and clinical data from the same patient group remain scarce. This discontinuity underscores one of the main issues of creating strong multimodal AI systems in leukemia studies. It is thus significant to understand the context of the use of these heterogeneous data types in artificial intelligence modeling by understanding how they come into existence in the clinical diagnostic workflow.

### 3.4 Leukemia diagnostic pipeline and data modalities

**Figure 2:**
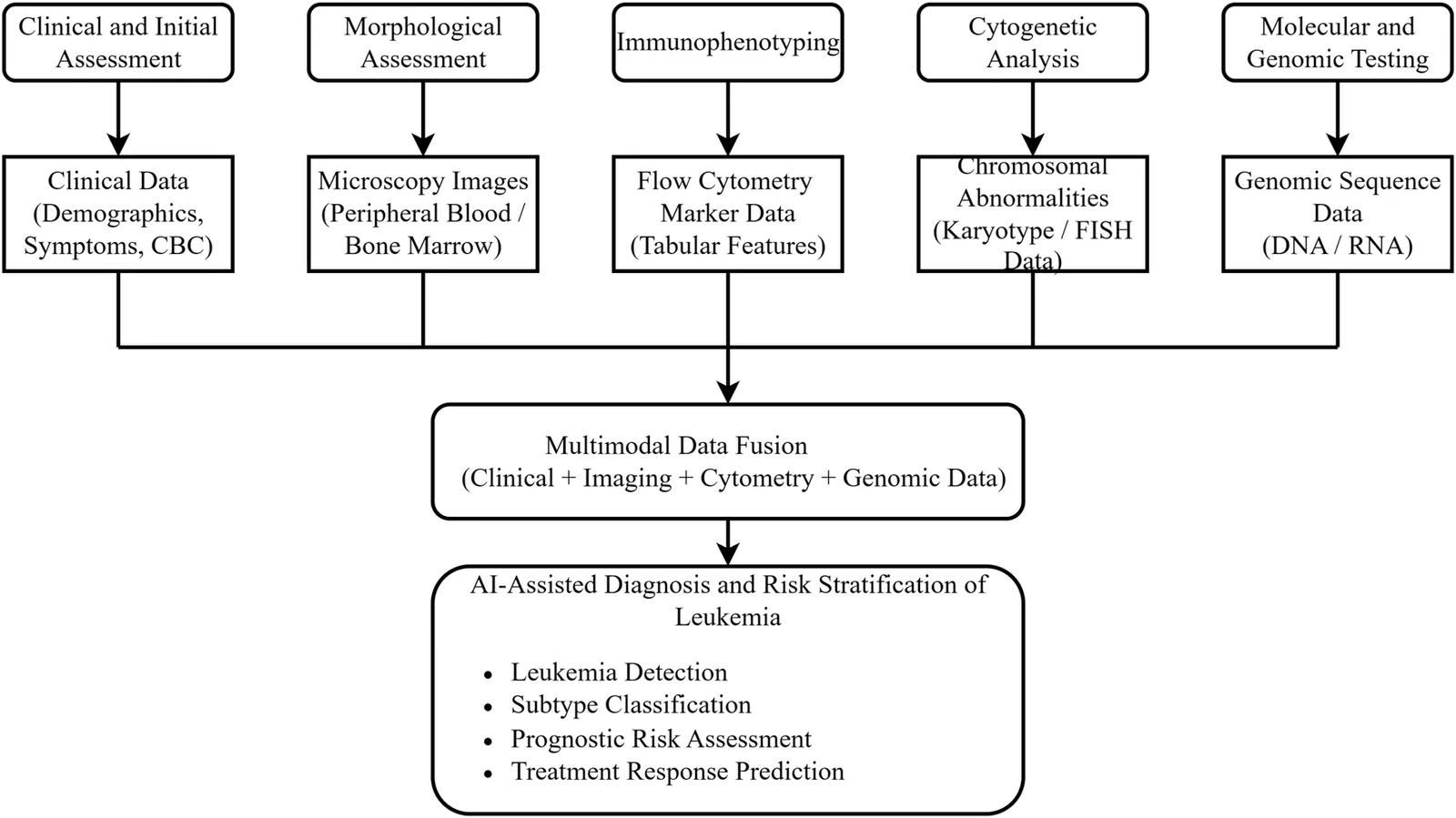
Clinical leukemia diagnostic workflow and associated data modalities.

To contextualise these heterogeneous datasets into perspective, Figure 2 shows the conventional diagnostic pathway of leukemia, starting with clinical examination and moving on to morphological examination, immunophenotyping, cytogenetic examination, and molecular genomic examination. The data modalities generated by each diagnostic stage are clinical records, microscopy images, flow cytometry marker profiles, chromosomal abnormality data, and genomic sequencing data. All these heterogeneous data sources are used to provide integrated diagnosis and risk stratification based on modern classification systems, such as the WHO and ICC frameworks. These modalities are important inputs in artificial intelligence research to create models to detect leukemia, classify its subtypes, and predict its prognosis. Microscopic imaging of peripheral blood and bone marrow samples is one of the most widely studied data sources for artificial intelligence-based leukemia detection and classification. As a result, many recent works have investigated deep learning models to detect leukemia and automatically classify morphological features of hematological images.

### 3.5 Deep learning approaches in leukemia imaging

Peripheral blood and bone marrow smears microscopic analysis is an essential part of the diagnosis of leukemia, which offers essential morphological clues, including blast morphology, nuclear chromatin patterns, and cytoplasmic features. Manual interpretation is, however, labor-intensive and prone to inter-observer variability. Recent progress in artificial intelligence, especially deep learning, has increased the pace of automated hematological image analysis systems that can perform tasks such as single-cell segmentation to leukemia subtype classification. This part summarizes AI methods in leukemia research based on Convolutional Neural Network (CNN)-based architectures to learn morphological features, transformer-based architectures to learn global contextual features, Explainable AI (XAI) to enhance interpretability, and data-efficient learning paradigms to overcome the problem of small annotated datasets.

#### 3.5.1 CNN-based architectures and transfer learning

Convolutional Neural Networks (CNNs) are the most popular architecture of automated hematological microscopy image analysis. Their hierarchical feature-learning ability enables them to extract complex morphological features, such as nuclear chromatin patterns, cytoplasmic granularity, and nucleus-cytoplasm ratios, which are essential for detecting leukemic blasts. As a result, CNN-based methods have been extensively used in imaging problems such as leukocyte detection, blast classification, and prediction of leukemia subtypes using peripheral blood and bone marrow smear images.

The current CNN-based leukemia imaging systems can be broadly divided into three methodological groups: (i) Detection-driven frameworks of leukocyte localization, (ii) CNN classifiers of blast and subtype identification, and (iii) Transfer-learning and hybrid CNN machine learning models that are aimed at limited medical datasets.

Detection-based pipelines are more commonly based on CNN-based feature extractors that are combined with object detection systems to localize individual leukocytes before classification. Faster R-CNN Two-stage detectors like Faster R-CNN, where the feature extraction is done using deep convolutional backbones and then the region proposal networks are used, are often used in the analysis of microscopic blood smears because they are highly localized in dense cellular regions. Conversely, one-stage detectors like YOLO offer faster inference and can be used in high-throughput screening systems (27–29). These detection systems allow automatic localization of leukocytes and abnormal cells in microscopic images, which is the initial step in most computer-aided leukemia diagnosis pipelines.

However, the detection models that have been trained on a single dataset may have a reduced performance when applied to the images acquired under different staining protocols or imaging platforms, which highlights the issue of staining variability and dataset bias in hematological image analysis.

Besides leukocyte detection, CNN models are also widely used for blast classification and leukemia subtype classification. Deep networks like ResNet, VGG, and DenseNet are trained to learn discriminative morphological representations that enable accurate classification of lymphoblasts and myeloblasts, which can be used to automatically classify ALL and AML (6, 30, 31). Ensemble CNN methods also enhance classification performance by combining predictions from multiple architectures, thereby reducing misclassification between morphologically similar lymphoblast subtypes (32, 33).

However, these classifiers are usually at the cell or image level, whereas clinical diagnosis involves combining results from many cells and smears.

Nevertheless, the scarcity of labeled hematological data is one of the primary challenges of deep learning model training. Transfer learning has therefore emerged as a popular approach to CNN-based leukemia classification. AlexNet, ResNet and other CNN backbones can be trained on blood smear images to learn leukemia-specific morphological features with less training data (34, 35). CNNs can also be applied as feature extractors on small datasets, and the final classification step is performed by classical machine learning classifiers, such as support vector machines, random forests, or gradient boosting (36).

To further enhance robustness, preprocessing methods, including stain normalisation and massive data augmentation, are incorporated in many studies to reduce variability caused by various staining protocols and imaging conditions.

CNN-based leukemia imaging systems have several challenges despite their good performance. Most of the studies are based on small or single-centre data, which restricts generalization across institutions. Moreover, staining, and inter-laboratory variations can be significant sources of poor model performance. Lastly, many CNN models do not examine patient-level morphology but isolated cells, which limits their direct clinical use. Although CNN-based architectures are still the most popular models in the analysis of hematological images, they are based on localized convolutional operations, which may restrict the ability to model long-range spatial dependencies. These constraints have led to the investigation of transformer-based architectures of leukemia imaging.

#### 3.5.2 Vision transformers and attention-based models

Although CNN models have demonstrated high standards in imaging of leukemia, the modeling of long-range spatial properties, such as the correlation between distant cytoplasmic granules and nuclear edge structures, may be limited by the nature of the CNN model, which has local receptive fields. To overcome these limitations, Vision Transformer (ViT) models and CNN-Transformer hybrid models have been developed, which use self-attention to learn global contextual relationships between image regions that are far apart (37).

Transformer-based models, unlike the sliding-window method of CNNs, split an image into discrete patches and process them sequentially to maintain a global field of view. Transformer-based architectures have demonstrated potential in hematological image analysis because they can capture global contextual relationships between image regions with self-attention mechanisms, which can better represent complex morphological patterns in blood cell images (38). Moreover, hybrid CNN Transformer models are being investigated to integrate local convolutional feature extraction and global contextual modelling (39).

Nevertheless, one of the significant drawbacks of transformer-based models in leukemia imaging is that they have large data and computational demands. Due to the limited size of annotated hematological datasets, recent trends in research focus on data-efficient transformer models, such as self-supervised learning and foundation models that can use large sets of unlabeled microscopic images to enhance feature representation and generalization (40).

#### 3.5.3 Explainable artificial intelligence (XAI) in leukemia imaging

Even though transformer-based architectures improve contextual representation in leukemia imaging, their complicated architectures tend to decrease interpretability. In turn, Explainable Artificial Intelligence (XAI) methods have become increasingly popular for providing visual explanations of deep learning predictions and enhancing transparency in clinical decision-support systems.

Several post-hoc interpretability methods have been used to study model behavior in hematological image analysis. Visualization methods based on Class Activation Mapping (CAM), like Grad-CAM, Score-CAM, and Eigen-CAM produce heatmaps that indicate diagnostically relevant morphological features in microscopic images, allowing researchers to determine whether deep learning models are targeting diagnostically relevant morphological features like nuclear contours, chromatin texture, and cytoplasmic granularity in leukemic blast cells (34, 35). More recent hybrid deep learning models that combine convolutional neural networks with transformer models have also added explainability methods like Grad-CAM, LIME, Integrated Gradients, and SHAP to indicate morphological regions that contribute to leukemia detection in peripheral blood smear images (41). Gradient-based explanation methods like Grad-CAM might, however, end up causing noisy or less faithful visualizations when used on a transformer-based architecture, where attention-based interpretability methods have been proposed to provide a better description of the token-level contribution.

XAI methods can be used to qualitatively validate computer-aided leukemia diagnosis systems by offering visual evidence of automated predictions, and enhance the confidence of clinicians in AI-assisted decision- making. New explainable ensemble models also use deep neural networks with interpretability methods to provide transparent classification results and minimize false predictions in leukemia screening systems (42).

However, the majority of existing XAI methods provide rough explanations of deep neural network behaviour and may not capture the intricate decision-making processes that underlie model predictions. The current research directions thus examine naturally interpretable architectures that incorporate attention mechanisms or segmentation-aware modules to identify diagnostically significant regions during model inference, thereby minimising the reliance on purely post-hoc explanation mechanisms. The creation of clinically interpretable and quantitatively reliable explanation frameworks is also a significant research direction towards reliable AI-based leukemia diagnostic systems.

#### 3.5.4 Critical synthesis: challenges and future directions

Although CNN- and transformer-based architectures show promising results in the analysis of leukemia images, several challenges remain on the way to reliable clinical translation:

- Low cross-institution generalization: The models trained on data sets collected under laboratory conditions can demonstrate lower performance when it comes to working with images captured with the help of other staining protocols or imaging devices.
- Minimal contextual information: Most existing methods use cropped single-cell images or small patches, which may not reflect the broader context of the smear that hematopathologists examine when manually evaluating it.
- Incomplete diagnostic representation: Morphological analysis cannot be used to diagnose leukemia fully, and it is usually necessary to combine it with immunophenotyping, cytogenetic results, and molecular changes.

To overcome these limitations, more robust models that can learn from larger multi-institutional datasets need to be developed, and diagnostic AI systems that integrate imaging features with molecular and clinical data should be designed.

In general, imaging-based methods are the most widely studied modality in leukemia AI research, primarily due to access to annotated blood smear datasets and advances in deep learning architectures. CNN and transformer-based models have shown encouraging diagnostic performance. Still, differences in datasets, image acquisition conditions, validation methods, and other factors make it difficult to compare across studies and could hinder clinical translation.

The following section discusses how deep learning approaches are applied to molecular and genomic data in leukemia research.

### 3.6 Deep learning for molecular and genomic analysis in leukemia

Although AI systems based on imaging offer high-throughput automated cellular morphology analysis, they mainly detect the phenotypic characteristics of leukemia. These visual characteristics are not always adequate to detect the subtype of the disease in modern clinical practice, with genetically distinct leukemias having very similar morphological appearances. Therefore, current diagnostic paradigms are increasingly reliant on molecular and genomic profiling to reveal the underlying mechanisms of leukemogenesis. Computationally, this shift necessitates a change in spatial feature extraction to architectures that can model high-dimensional transcriptomic, mutational, and multi-omics data, where the number of patient samples (N) is significantly less than the number of molecular features (P) (N≪P). To overcome this problem, recent works have considered more sophisticated deep learning models, such as latent representation learning with autoencoders to reduce dimensions, Graph Neural Networks (GNNs) to model gene interaction networks, and transformer-based models for sequence-aware genomic modeling. The approaches aim to extract informative molecular signatures from large-scale transcriptomic data to identify leukemia subtypes, biomarkers and prognosis (9). However, molecular AI systems can address several issues, such as high-dimensional data representations, batch effects across sequencing platforms, and class imbalance in rare leukemia subtypes.

#### 3.6.1 Representation learning for high-dimensional transcriptomic data

Microarray and RNA sequencing (RNA-seq) are high-throughput transcriptomic technologies that generate gene expression data with thousands of molecular features, which are a challenging environment for predictive modeling. In such environments, conventional feature engineering approaches struggle to learn complex nonlinear gene-gene interactions, particularly when the available patient sample is small. To address this, recent research has turned to increasingly popular deep learning models for representation learning, which can learn latent representations from high-dimensional molecular features.

Autoencoders (AEs) and their variants are a successful approach to learning efficient representations of transcriptomic data. The models have an encoder-decoder structure that transforms gene expression data into lower-dimensional vectors that encode the biological profile of interest while minimising non-informative variance and noise. In particular, Stacked Denoising Autoencoders (SDAEs) enhance representations by training feature abstractions in a hierarchical manner via layer-wise denoising. These deep architectures are capable of learning nonlinear patterns of gene co-expression that are related to the heterogeneity of leukemia subtypes and disease progression, which is superior to the traditional linear dimensionality reduction algorithms like Principal Component Analysis (PCA) (43).

The trained latent embeddings may then be used as powerful inputs to predictive models. Deep learning based feature representations have been used in several leukemia transcriptomic studies, where they are combined with existing machine learning classifiers such as support vector machines and random forests, to create hybrid or ensemble models that outperform in high-dimensional, small-sample gene expression data. These representations are used together with Multi-Layer Perceptrons (MLP) or ensemble classifiers in most studies to classify leukemia subtypes, identify biomarkers, and risk-stratify patients (44). More recent deep learning models that combine optimized feature selection and neural networks have also shown high predictive accuracy on leukemia subtype classification using high-dimensional gene expression data (45). Representation learning frameworks enable processing high-dimensional transcriptomic data by collapsing decentralised features into compact representations, and thereby achieving model stability and predictive accuracy, especially when clinical datasets have fewer annotated samples.

#### 3.6.2 Relational modeling via graph neural networks (GNNs)

Although latent representation techniques are effective at compressing gene expression data, they generally assume that genes are independent features, thereby ignoring the fact that biological systems are inherently interconnected. In reality, genes are involved in regulatory, signaling and Protein-Protein Interaction (PPI) networks. These topological interactions can be neglected, potentially reducing a model’s ability to explain the mechanisms of leukemogenesis. To address this limitation, Graph Neural Networks (GNNs) are appealing models for molecular data analysis, as they integrate prior knowledge of the system into the learning process.

In genomic contexts, one can model genes as nodes in a graph and known biological interactions as edges between them, such as signalling pathways, regulatory relationships, or co-expression relationships. GNN models, and especially Graph Convolutional Networks (GCNs) and Graph Attention Networks (GATs), transmit information through these networks via message-passing mechanisms where each gene node can receive contextual information about its neighbors (46). As a result, the acquired embedding of a gene not only indicates the value of its expression but also the functional status of its interacting partners. By combining high-dimensional transcriptomic profiles with known biological interaction networks, GNN-based models offer a biologically informed inductive bias, thereby enhancing predictive and interpretable performance. Recent research has shown that these network-sensitive deep learning models can improve activities such as cancer driver gene discovery, biomarker discovery, and pathway-scale analysis in large-scale genomic data (47).

#### 3.6.3 Transformer-based architectures for genomic modeling

Although Graph Neural Networks (GNNs) leverage existing biological interaction networks, their performance is necessarily limited by the incompleteness of pathway annotations and interactome databases. Transformer-based architectures have recently emerged as powerful deep learning models capable of learning complex dependencies in high-dimensional genomic data, thereby overcoming these limitations. Using self-attention methods, transformers can capture long-range relationships in genomic sequences without requiring explicit prior network structures. Recent transformer-based genomic language models have been shown to learn contextual representations of DNA sequences and regulatory elements directly from large-scale genomic data and to predict functional genomic features, including promoter activity, enhancer elements, splice sites, and chromatin profiles, with accuracy across a wide range of biological tasks (48).

Based on these advances, more recent progress in genomic foundation models, such as transformer-based models trained on large-scale genomic and transcriptomic data, has further increased the potential of this method. Other models like the Nucleotide Transformer are trained on thousands of human genomes and across several species, allowing the context-sensitive representations of nucleotide sequences to be learned by self-supervised training goals like masked language modeling (49).

These learned representations may then be refined to downstream genomic activities such as splice-site, enhancer prediction, variant prioritization and mutation classification. These types of transfer learning have found application especially in the case of leukemia where accessible genomic data are frequently small and highly heterogeneous across disease subtypes. In addition, transformer-based models such as DNABERT-style models have been demonstrated to be able to identify mutation patterns and classify genomic variants in cancer data, which indicates that they can be used for leukemia genomics and precision medicine (50).

Despite their promise, transformer-based genomic models face several challenges. Normal self-attention methods have quadratic computational complexity with respect to sequence length, which is prohibitive for computing genome-scale datasets with thousands of genes. Moreover, the large transformer architecture training is often computationally expensive and needs large datasets, which can be a limiting factor in its immediate use in leukemia-specific genomic research where data is often limited. Furthermore, the biological meaning of attention weights remains a significant interpretive challenge, as the translation of model attention patterns into clinically relevant molecular information must be cautiously combined with current biological knowledge and experimental evidence.

These limitations highlight the need for methods that increase the readability and understanding of deep learning models used in molecular diagnostics. As a result, Explainable Artificial Intelligence (XAI) methods have become a significant trend in the interpretation of the biological nature of model predictions in leukemia genomics.

#### 3.6.4 Explainable AI for molecular diagnostics

With the growing use of deep learning models to detect leukemia and subtype classification using genomic and transcriptomic data, there has been a need to understand the molecular characteristics underlying model predictions to enable clinical translation. Explainable Artificial Intelligence (XAI) methods aim to provide insights into the decision-making of complex models by identifying the genes, mutations, or regulatory factors that contribute most to the diagnostic predictions.

Deep learning models trained on leukemia gene expression data have been used with explainable artificial intelligence methods like SHapley Additive Explanations (SHAP) to determine the most important biomarker genes that lead to predictive results. As an example, deep learning models combining autoencoder-based dimensionality reduction with neural network classifiers and SHAP-based feature attribution have been applied to high-dimensional gene expression data in chronic lymphocytic leukemia. These methods allow the discovery of influential genes, such as CEACAM19, PIGP, MKL1, and IGF1R, which demonstrate that XAI methods can be used to identify biologically significant molecular signatures underlying predictive models and aid in the discovery of biomarkers in leukemia genomics (49).

In addition to deep learning models, several studies have investigated explainable artificial intelligence methods to enhance the interpretability of molecular diagnostic models. SHAP-based feature attribution models have been applied to determine the important biomarker genes in leukemia by measuring the impact of each gene feature on model predictions (51).

#### 3.6.5 Critical synthesis: challenges and future directions

Although recent developments in deep learning methods have been made in the analysis of genomics and transcriptomics in leukemia, there are still several challenges that restrict their clinical implementation

- High-dimensional molecular data: Genomic and transcriptomic data sets have thousands of features and relatively small cohorts of leukemia, which increases the chances of overfitting the model.
- Restricted size of annotated datasets: Model training or validation is restricted by the size of available large and well-annotated leukemia molecular datasets.
- Limited multimodal integration: Most studies are based on single-modality datasets, whereas clinical leukemia diagnosis requires integrating genomic, transcriptomic, and clinical information.
- Interpretability challenges: Even though XAI tools like SHAP and gradient-based attribution algorithms can identify genes that have an impact, it is challenging to associate the results with biologically relevant processes.
- Computational complexity: The more sophisticated models, like transformer-based models, demand high computational resources and huge training data.

To overcome these issues, data-efficient learning methods, enhanced multimodal combination of molecular data, and more biologically interpretable AI models to diagnose leukemia need to be developed.

Overall, genomic and transcriptomic methods provide relevant information about the molecular diversity of leukemia and help identify leukaemia subtypes, assess prognosis, and discover biomarkers. Various methods have been proposed and shown to be effective for learning complex molecular representations, including autoencoders, Multi-Layer Perceptrons (MLPs), Graph Neural Networks (GNNs), Graph Attention Networks (GATs), and transformer-based models. However, due to variation in sequencing platforms, patient cohorts, data processing and feature representation techniques, and validation approaches, it is difficult to directly compare results across studies. It may hinder the ability to reproduce and translate these findings into clinical practice.

### 3.7 Deep learning for clinical and flow cytometry data

Although molecular and genomic profiling offer important information on the biological processes and genetic changes that cause leukemia, standard clinical laboratory studies and immunophenotypic studies are still important parts of diagnostic procedures in hematology. In clinical practice, the first suspicion of leukemia is usually based on the abnormalities of the routine hematological parameters, and the confirmatory tests are performed with the help of such methods as multiparametric flow cytometry (1). These data sources have multidimensional patterns that may be difficult to analyze with traditional methods of analysis. As a result, recent studies have investigated how machine learning and deep learning methods can be used to derive diagnostically relevant information using clinical laboratory measurements and cytometric data (4, 52).

Clinical laboratory and flow cytometry data are more readily available and generated as part of routine diagnostic workups, unlike genomic tests, and are attractive targets for data-driven diagnostic decision support systems. Recent work suggest that machine learning approaches can be applied to routine hematology data to predict leukemia subtypes, and deep learning approaches have demonstrated effectiveness in automated analysis of high-dimensional flow cytometry data to detect and classify leukemia (4, 53). The following subsections give an overview of recent developments in deep learning approaches to clinical laboratory data and flow cytometry measurements analysis in leukemia research and diagnostics.

#### 3.7.1 Deep learning for clinical laboratory data

Machine learning and deep learning models trained on large clinical datasets have indicated promising results in predicting the type of leukemia based on routine hematological measurements of hemoglobin, platelets, leukocytes and other laboratory tests. These models can be applied to rapidly assess preliminary risks and may help in early diagnosis using standard clinical measurements (4).

Recent articles have examined machine learning strategies to examine regular clinical laboratory parameters to predict early leukemia subtypes. An extreme gradient boosting model was trained on routine blood test variables from more than 1,400 patients to differentiate between AML, ALL, and APL. The model showed good diagnostic performance and emphasizes the possibility of using laboratory data that is readily available to conduct preliminary screening. Nevertheless, the method relies primarily on tabular laboratory variables and lacks morphological or molecular data that could further improve diagnostic performance (4).

Hybrid deep learning and machine learning frameworks have also been studied recently to analyze routine hematological data. In another study, convolutional neural networks were applied to extract morphological features of white blood cell scattergrams, which were then combined with standard blood cell parameters to build a random forest classifier to screen acute promyelocytic leukemia. This framework demonstrated high diagnostic accuracy (AUC > 0.98) and can be used to screen quickly using routinely generated laboratory data. However, these findings should be interpreted in the context of the study cohort, sample size, and validation strategy, and further prospective validation is needed to establish real world generalizability. Furthermore, the model is mainly intended to be used in APL detection and can rely on the presence and standardization of hematology analyzer scattergram data in clinical environments (54).

The use of deep learning and machine learning models to analyze clinical laboratory parameters and electronic health records in leukemia prediction tasks has also been investigated. Neural network models trained on clinical and laboratory data have shown promise for predicting disease outcomes and patient survival from routine medical data, but are less widespread than deep learning models based on imaging.

#### 3.7.2 Deep learning for flow cytometry analysis

Multiparametric flow cytometry is commonly applied in immunophenotypic characterization of leukemic cell populations. Nevertheless, the traditional analysis relies on manual gating plans and expert interpretation, which may introduce subjectivity and variability across laboratories. New deep learning methods have thus been developed to automate the analysis of high-dimensional cytometry data and enhance diagnostic efficiency.

A recent study suggested an attention-based deep learning model to analyze flow cytometry data in the diagnosis of leukemia. The model examined large groups of clinical cytometry samples and performed well in the detection of acute leukemia and the differentiation of AML and ALL. In particular, the approach demonstrated the ability to predict cytogenetic abnormalities and molecular variants from immunophenotypes, suggesting that deep learning may link cellular phenotypes to genetic alterations. One of the primary benefits of this method is that it eliminates manual gating through automated identification of cell populations of interest. Nevertheless, the model was constructed from a single cytometry panel, which may limit its extrapolation to other institutions with different marker settings (52).

Another deep learning method used neural network structures to directly classify leukemia using multiparametric flow cytometry measurements. The model proved to be very accurate in the detection of leukemic cell populations and the differentiation of the major disease subtypes like AML and B-cell acute lymphoblastic leukemia. These results indicate that deep learning models have the potential to learn intricate associations between cytometry markers and to make rapid, automated diagnostic predictions. However, the research primarily concentrated on the classification performance, and additional validation on multi-centre datasets and rare leukemia subtypes is yet to be done (53).

In general, these studies show that deep learning has the potential to revolutionize flow cytometry analysis into an automated and reproducible diagnostic workflow in hematology laboratories.

#### 3.7.3 Explainable AI for clinical and cytometry-based decision support

Although explainability methods are common in imaging and genomic models, their use in clinical laboratory and flow cytometry data is mainly aimed at explaining structured clinical variables and immunophenotypic marker patterns. Explainable Artificial Intelligence (XAI) methods, in that case, seek to determine which laboratory parameters and cytometric features underlie model predictions, thus assisting clinicians in interpreting them and integrating AI-based systems of decision support into the workflow of hematology (13, 42).

#### 3.7.4 Critical synthesis: challenges and future directions

Among the 59 included studies, 32 focused on microscopic imaging, 20 on genomic or molecular data, 3 on flow cytometry, and 3 on clinical laboratory data. Fewer studies were identified for clinical laboratory and flow cytometry applications in leukemia diagnostics than for imaging and genomic studies. This imbalance may be due to publicly available imaging datasets and established genomic datasets. In contrast, clinical laboratory and flow cytometry data may be more difficult to standardize, interoperate with, share, and make publicly available. Furthermore, the increased focus on imaging-based AI applications in research could have led to an increased number of imaging studies being published recently.

Although recent advances have been made in the application of machine learning and deep learning to clinical laboratory and flow cytometry data to diagnose leukemia, there are several challenges that restrict their clinical implementation:

- Limited dataset size and diversity: A lot of the current literature uses relatively small datasets or single-centre datasets, which can decrease the applicability of the developed models to a wide range of leukemia patients.
- Data heterogeneity across laboratories: Differences in the measurements of hematology laboratories, immunophenotyping panel flow cytometry and instrument settings may influence the strength and reproducibility of AI models across laboratories.
- Limited use of advanced deep learning models: The majority of clinical laboratory research still relies on conventional machine learning approaches on tabular data, and more complex deep learning and transformer models are not widely used in clinical and cytometric data analysis in leukemia research.
- Challenges in model interpretability and clinical validation: While representation learning and explainable AI have improved the understanding of clinical factors and immunophenotypic markers, further validation is required to ensure that these models can provide meaningful and accurate decision support.

To address these challenges, we need to build large, multi-site cohorts of leukemia, standardize laboratory and cytometry data collection, and develop multimodal learning systems that integrate clinical, cytometric, imaging and genomic data to build more accurate and clinically useful AI-based diagnostic systems.

In general, clinical laboratory and flow cytometry-based approaches provide useful data for leukemia screening, diagnosis, classification and risk assessment. Machine learning and deep learning models have shown promising results using routine hematological parameters, electronic health records, and high-dimensional immunophenotypic data, suggesting their potential for clinical decision-making. However, direct comparisons between studies are difficult due to differences in patient populations, data quality, instrumentation, data preprocessing methods, and validation methods, which may limit the reproducibility and clinical translation of these results.

### 3.8 Overview of deep learning architectures across leukemia data modalities

In leukemia studies, a wide range of deep learning architectures have been used across various biomedical data modalities, as summarised in Table 2. Convolutional neural networks are primarily used for morphological image analysis, whereas transformer-based and autoencoder models are commonly used for high-dimensional genomic data. Structured learning methods or neural networks that are modified to work with tabular data are commonly used to model clinical and flow cytometry data. Although these developments have been made, most of the current research is on single-modality analysis, which only provides a partial picture of leukemia biology. Since clinical diagnosis is usually based on the synthesis of morphological, immunophenotypic, molecular, and clinical data, recent investigations have focused on multimodal artificial intelligence systems that integrate heterogeneous data streams. Section 3.9 discusses key multimodal fusion approaches suggested in the diagnosis of leukemia.

**Table 2:** Deep learning architectures across leukemia data modalities.

Data modality	Architecture(s)	Primary application	Technical scope	References
Microscopic Imaging	CNN (ResNet, DenseNet, EfficientNet), YOLO	Blast cell detection and morphological screening	Local spatial feature extraction and object detection	(5, 6, 8, 29, 30)
Microscopic Imaging	Vision Transformer, Swin Transformer	Fine-grained leukemia cell classification	Global contextual modeling using self-attention	(17, 38)
Genomic or Molecular	Transformer-based models	Gene mutation analysis and sequence modeling	Self-attention modeling of genomic sequences	(48–50)
Genomic or Molecular	Deep neural networks, autoencoder-based models	Biomarker discovery and gene expression classification	Latent feature learning in high-dimensional omics data	(43–45)
Clinical Laboratory Data	Machine learning and neural networks	Prognostic modeling and leukemia subtype prediction	Structured tabular feature modelling	(4)
Flow Cytometry	Deep learning models	Automated immunophenotyping and leukemia detection	High-dimensional cellular feature aggregation	(13, 52, 53)

Even though these architectures have shown good performance on single data modalities, clinical practice in leukemia diagnosis requires combining morphological, molecular, immunophenotypic, and laboratory data. As a result, recent research has shown growing interest in multimodal artificial intelligence systems that integrate heterogeneous biomedical data sources.

### 3.9 Multimodal artificial intelligence frameworks for leukemia diagnosis

Although unimodal deep learning models have made considerable progress, such as microscopic imaging, genomic profiling, and clinical laboratory measurements, methods that are limited to one type of data only reveal part of the picture in leukemia diagnosis. Recent research has shown that machine learning can be applied to individual modalities, including clinical laboratory parameters, multi-omics molecular profiling, and flow cytometry analysis (4, 12, 52, 53). Nevertheless, independent analysis of these data sources can limit the ability to uncover complex cross-modal relationships. In turn, recent work have been focusing more on multimodal artificial intelligence systems that combine heterogeneous biomedical data to enhance the accuracy of diagnosis and clinical decision support in leukemia.

There are typically a variety of different fusion strategies used by multimodal AI systems to integrate heterogeneous biomedical data, which may be loosely classified into early fusion, intermediate or joint fusion, late fusion, and cross-modal attention-based architectures.

**Figure 3:**
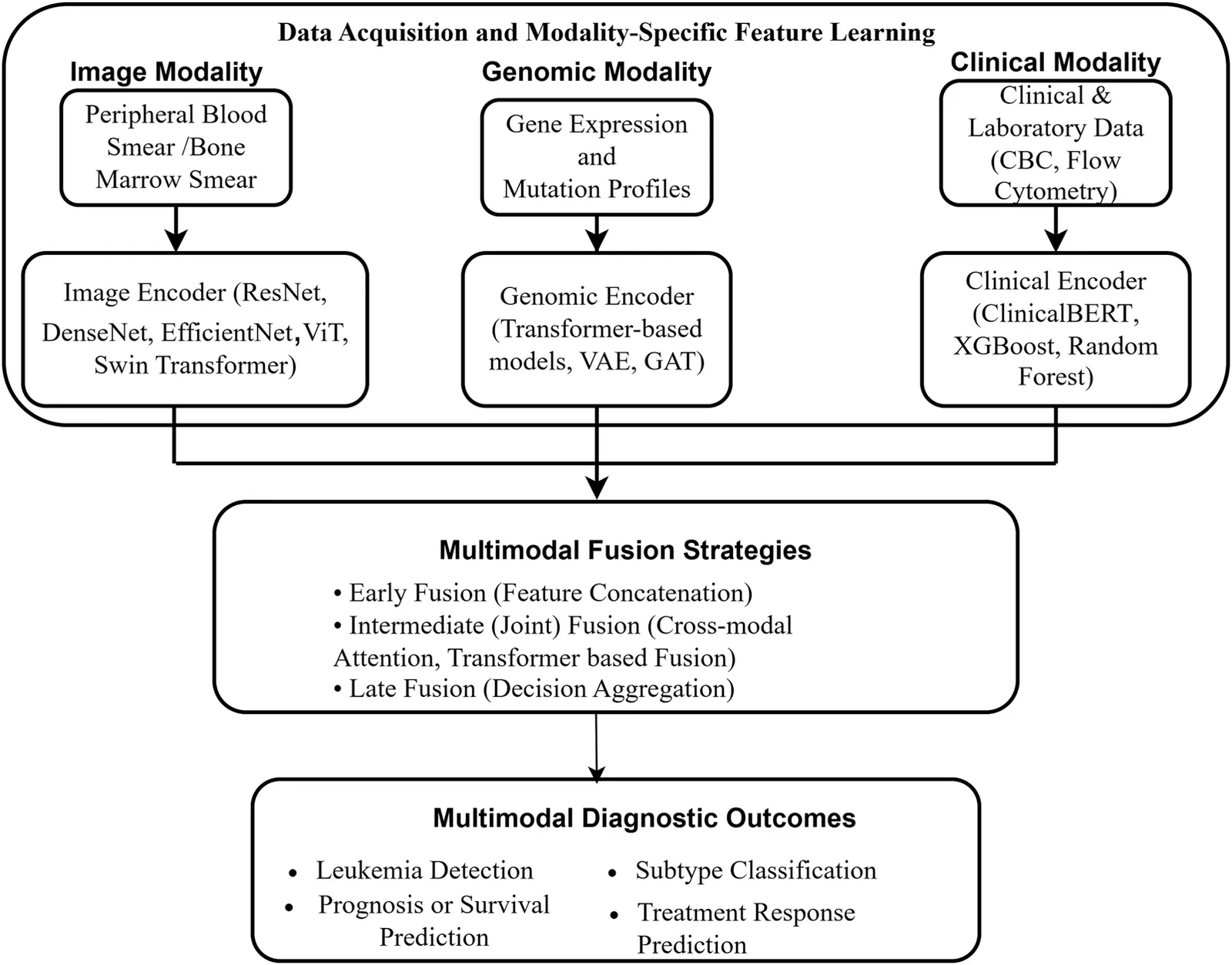
Conceptual framework illustrating a potential multimodal artificial intelligence system integrating imaging, genomic, and clinical data for leukemia diagnosis.

As illustrated in Figure 3, multimodal AI systems typically follow a hierarchical pipeline that includes modality-specific feature extraction, representation learning, and multimodal fusion. Imaging data are processed using convolutional or transformer-based architectures, while genomic and molecular data are modeled using transformers, autoencoders, or graph-based approaches. Clinical and laboratory parameters are analyzed using models designed for structured data. The resulting representations are then integrated through different fusion methods to support diagnostic decision-making.

#### 3.9.1 Early fusion strategies

Early fusion methods combine heterogeneous feature representations at the input or feature level prior to model training, enabling joint representations to be learned across multiple sources of information. In the study of leukemia, feature-level fusion has been considered to improve the morphological characterization of leukemic cells. A multiscale CNN architecture called 3SNet combines grayscale leukemia images with complementary texture features like Histogram of Oriented Gradients (HOG) and Local Binary Patterns (LBP), with the extracted features being combined to produce a more powerful representation of leukemia cells (55). Likewise, the XIncept-ALL model integrates deep features from various CNNs, such as InceptionV3 and Xception, by concatenating them before classification (56). These methods reveal that the combination of complementary feature representations can enhance the performance of leukemia detection and subtype classification. One benefit of early fusion is that it can capture more morphological patterns by combining multiple feature descriptors. Nevertheless, many current implementations are based on image-derived features and lack the inclusion of other clinical or molecular modalities, which can be a limiting factor in their capacity to capture the biological complexity of leukemia.

#### 3.9.2 Intermediate (joint) fusion approaches

The Intermediate or joint fusion approaches combine heterogeneous modalities within internal layers of a neural network and enable the model to learn joint latent representations that capture inter- and intra-modal interactions across multiple biological data sources. This paradigm has been studied in leukemia research in the context of integrating genomic and transcriptomic signals derived from single-cell RNA sequencing. Notably, a recent method, scVAR, uses a variational autoencoder to simultaneously learn transcriptomic and genetic variant data within a shared latent space (57). The model can better identify cellular subpopulations and capture fine genotype—phenotype interactions that may otherwise be missed in independent unimodal analyses by learning an integrated embedding space. An additional benefit of using this framework is that it allows for the integration of genomic and transcriptomic data derived from the same sequencing modality, providing a detailed view of the cellular heterogeneity of the leukemia cells. Another important advantage of joint fusion models is their capacity to reveal cross-modal biological associations and improve the identification of disease-related cellular states. Nevertheless, one major limitation is that these models rely on high-quality single-cell sequencing data and can be affected by data sparsity and uneven coverage in scRNA-seq datasets.

#### 3.9.3 Late fusion models

Late fusion works at the decision level and combines the predictions of independently trained models, such that each model learns to specialize in the representation of features and then combines their outputs to make the final diagnosis. Ensemble-based late fusion frameworks have been shown to have better classification robustness in leukemia research through the combination of predictions made by multiple deep learning models. In particular, the Leuk-XAI-EDL model, which combines the outputs of multiple convolutional neural networks using ensemble techniques to enhance leukemia classification performance (42). In the same manner, an ensemble diagnostic model was created that integrates deep features obtained using DenseNet-121 and ResNet-34 with various machine learning classifiers, with the final prediction being achieved by classifier ensemble voting (58). The primary value of these studies is that they demonstrate that integrating complementary models at the decision level can increase diagnostic reliability and reduce model bias. One of the benefits of late fusion is its flexibility in combining heterogeneous models without requiring unified feature representations. Nevertheless, one limitation is that inter-modal or cross-feature relationships might not be fully represented, since interactions between data sources are learned only during independent model training.

#### 3.9.4 Cross-modal attention architectures

Cross-modal attention architectures are a more recent development in multimodal artificial intelligence, in which transformer-based systems are applied to simulate interactions between heterogeneous biomedical modalities. Such methods have been suggested in the diagnostics of leukemia to combine morphological image data with clinical textual data to more accurately represent the multimodal reasoning of hematologists in the diagnosis process. As an illustration, a recent work proposed an explainable federated transformer architecture, which integrates a Vision Transformer to encode hematological smear pictures and ClinicalBERT to extract semantic features of clinical reports (59). The derived embeddings are then aligned with a cross-modal fusion layer, which allows simultaneous prediction of the presence and the stage of the disease in leukemia in a federated learning system to maintain the privacy of patient data across organizations. The primary value of this framework lies in combining multimodal transformer encoders with a privacy-sensitive federated training framework, enabling collaborative model learning without exchanging sensitive clinical information. The main benefit of this method is that it can obtain complementary diagnostic cues of both visual and textual modalities and make interpretable predictions using explainable AI methods. However, due to the architecture, more computational complexity is introduced, and coordinated multimodal data between the involved institutions may be necessary, which can be detrimental to real-world large-scale applications.

All these multimodal fusion strategies provide complementary approaches for combining heterogeneous biomedical data sources in leukemia diagnosis. Such systems can capture complex cross-modal relationships that may remain unnoticed when only a single modality is used. The overall workflow of multimodal artificial intelligence systems for leukemia diagnosis can be conceptually summarized as follows (Algorithm 1).

Algorithm 1 **Conceptual Multimodal Artificial Intelligence Workflow for Leukemia Diagnosis.**

**Require:** Multimodal biomedical data, including peripheral blood smear images, genomic profiles, and clinical laboratory parameters.

**Ensure:** Predicted leukemia subtype, treatment response estimation, and prognostic risk stratification

1: **Data Acquisition:** Multimodal data are obtained from clinical and laboratory data.
2: **Modality-Specific Preprocessing and Feature Extraction:**
  (a) Convolutional neural networks or vision transformers are used to extract features from microscopic images.
  (b) Genomic profiles are encoded using transformers, autoencoders or graph neural networks.
  (c) Data from clinical and laboratory tests are processed with machine learning or deep neural network algorithms.
3: **Feature Representation:** Each modality is represented by a feature embedding.
4: **Multimodal Feature Fusion:** Multimodal representations are combined using early fusion, intermediate (joint) fusion, late fusion, or cross-modal attention.
5: **Model Training and Prediction:** The multimodal representations are used to train predictive models.
6: **Diagnostic Output Generation:** Diagnostic results are generated, such as classification of the leukemia subtype, prediction of response to treatment, and risk of disease progression.

The above process can be expressed as learning feature representations for each modality and fusing them into a single predictive model. The first step is to learn a feature representation for each modality, which is then fused. The fused representation is then used to make predictions, and the learning process is usually guided by conventional loss functions, such as cross-entropy loss for classification and mean squared error for regression. Model performance is evaluated based on the clinical objective using commonly adopted metrics, including accuracy, F1-score, ROC-AUC, and concordance index.

#### 3.9.5 Critical synthesis and future research directions

Multimodal artificial intelligence systems have demonstrated growing capabilities of enhancing the diagnosis of leukemia through the combination of heterogeneous biomedical data, such as imaging, genomic profiles, and clinical laboratory data. Among the 59 included studies, only one study (59) used a true cross-modal fusion approach that combined different clinical data modalities. In contrast, (42, 58) employed ensemble or hybrid approaches involving multiple models or feature representations, whereas (57) integrated multiple genomic data types within the same sequencing modality rather than across different clinical modalities. Thus, the main finding of this study is that although multimodal fusion has the potential to represent real-world clinical decision-making better, it remains largely underexplored in leukemia diagnosis. This limited adoption may be due to the lack of publicly available same-patient multimodal datasets comprising blood smear images, genomic data, flow cytometry measurements, clinical laboratory data, and corresponding diagnostic labels. Nevertheless, there are still several challenges that restrict the practical use of these strategies.

- Limited availability of multimodal datasets: There are still limited large-scale datasets of leukemia that include imaging, genomic, and clinical data of the same patient, which limits the creation and testing of multimodal AI models.
- Complexity of heterogeneous data integration: The imaging, molecular, and clinical data vary significantly in terms of structure, scale, and dimensionality, and effective feature fusion and representation learning is technically difficult.
- Model interpretability challenges: Multimodal deep learning models tend to add complexity to the model, and it is harder to understand how various modalities are used to make the final diagnostic decision.

These issues have also been encountered in other multimodal AI applications across the wider field of oncology, where multimodal datasets are scarce and difficult to harmonize, and integrating clinical, imaging, and molecular information is complex (60).

To overcome these issues, there is a need to create interpretable multimodal architectures and to create multi-institutional data-sharing programs to enable large integrated datasets to enhance model generalization and clinical use. In addition to these challenges, there are several practical issues that have to be resolved in the implementation of the multimodal leukemia diagnostic systems. Correct modality alignment is crucial, as imaging, genomic, and clinical data need to be aligned at the patient level to achieve meaningful feature fusion. In real-world clinical settings, missing or incomplete modalities remain prevalent and can significantly impact model robustness. Additionally, multimodal architectures can be very resource-intensive, requiring significant computational power, storage space, and data management systems, which can limit their scalability and use in everyday clinical settings. There are also challenges in generalizing the models and translating them to the clinic due to differences in acquisition protocols and data quality between institutions.

## 4 Discussion

The recent developments in artificial intelligence have greatly changed the diagnosis of leukemia by allowing the automated processing of heterogeneous biomedical data. The reviewed studies in this survey show that deep learning models used on microscopic imaging have performed well in the detection of blast cells and the classification of leukemia subtypes. In a similar manner, deep learning architecture-based genomic and transcriptomic data analysis has demonstrated significant potential to discover alterations in the molecules, predict the pattern of gene expression, and enhance prognostic prediction in leukemia. Moreover, methods based on clinical laboratory parameters and flow cytometry data provide complementary data for disease monitoring and risk stratification. Although these unimodal methods have shown promising results, clinical diagnosis of leukemia is usually based on the combination of various complementary diagnostic modalities. In turn, recent studies have begun to consider multimodal artificial intelligence systems that integrate imaging, genomic, and clinical data to offer a more detailed picture of disease features and improve diagnostic decision support.

### 4.1 Comparative insights across data modalities

The artificial intelligence methods of leukemia diagnosis use various modalities of biomedical data, each of which offers different diagnostic data. Deep learning algorithms applied to microscopic images can automatically identify blast cells and morphological abnormalities in peripheral blood and bone marrow samples. Genomic and transcriptomic analyses can be used to detect mutation profiles and molecular markers associated with different types of leukemia, while clinical laboratory tests, such as complete blood count (CBC) tests, can be used for diagnostic prediction with machine learning. In addition, flow cytometry provides rich immunophenotypic information that is essential for the diagnosis of leukemia. While each of the modalities has its own merits, they all suffer from issues of data availability, dimensionality and standardization. Their diagnostic value, advantages and limitations are briefly outlined in Table 3. These limitations indicate the need for multimodal artificial intelligence systems that integrate complementary diagnostic information from multiple modalities for more accurate and reliable diagnosis of leukemia.

**Table 3:** Summary of recent deep learning studies for leukemia analysis across different data modalities, grouped by validation approach.

Related work	Modality	Model	Performance metrics	Limitations
Internal/single-institution validation				
(33)	Imaging	EfficientNet-V1/V2 Ensemble	Accuracy: 88.6%, F1: 0.736	∙ Class imbalance ∙ Single institution dataset
(32)	Imaging	Deep ensemble CNN (ResNet-152)	Accuracy: 99.95%	∙ High computational cost ∙ Single-source dataset ∙ 10-fold cross-validation only ∙ No external validation
(58)	Imaging	DenseNet-121 + ResNet-34 + ML ensemble	Accuracy: 92.5%, F1: 0.931, AUC: 0.975	∙ Dataset dependency ∙ High computational cost ∙ Lack of explainability ∙ Needs multi-center validation
(35)	Imaging	Transfer learning (VGG16, DenseNet121, MobileNet)	Accuracy: 84%	∙ Small dataset
(61)	Imaging	Hybrid CNN–ML models	Acc: 88%	∙ Small dataset ∙ Deeper model limitations
(30)	Imaging	RegNet CNN	F1: 0.762	∙ Class imbalance ∙ Rare cell classification difficulty
(62)	Imaging	ReLViT-GY-AML	Accuracy: 98.16%	∙ High computational cost ∙ Single dataset evaluation ∙ No external validation
External validation				
(2)	Imaging	Diffusion model (CytoDiffusion)	Accuracy: up to 0.9965	∙ High computational cost ∙ Dataset-dependent performance
(37)	Imaging	MultiPathGAN + MobileViTv2	Accuracy: 94.28%	∙ Limited dataset sources
(3)	Imaging	ResNet18 pipeline	Area Under the Receiver Operating Characteristic Curve(AUROC): up to 0.93	∙ Lower subgroup performance ∙ No multi-center validation
Internal/single-institution validation				
(63)	Genomic	Autoencoder + NN + SHAP	Accuracy: 86.36%	∙ Lack of external validation
(45)	Genomic	DDDQ-N	Accuracy: 99%	∙ Small dataset ∙ Generalization issues ∙ High computational cost
(64)	Genomic	GAN-U-Net + ROCKET	Accuracy: 99.26%	∙ Small dataset ∙ Synthetic data dependency ∙ Computational complexity
(65)	Genomic	TFAE + LightGBM	Accuracy: 0.986	∙ No external validation ∙ Requires biological validation
(66)	Genomic	ML + DL models	Accuracy: up to 98%	∙ Single dataset ∙ Simulation-based study
(57)	Genomic	scVAR (Variational Autoencoder with cross-attention multimodal integration)	Identified 20–30% more cellular subpopulations	∙ Limited genomic coverage in droplet-based scRNA-seq ∙ Sparse variant matrices
External validation				
(11)	Genomic	ML survival models	AUC: up to 0.925	∙ Reliance on public datasets ∙ No multi-center validation ∙ Interpretability issues
Internal/single-institution validation				
(67)	Clinical	Transformer survival model	Concordance Index(C-index): 0.945	∙ Small dataset ∙ Potential overfitting
Internal/single-institution validation				
(52)	Flow cytometry	ABMILM	AUROC: 0.961	∙ Single institution ∙ Limited mutation samples
(53)	Flow cytometry	ResNet-50 + EverFlow	Accuracy: up to 98.2%	∙ Small dataset ∙ Limited protocol
Internal/single-institution validation				
(59)	Multimodal	ViT + ClinicalBERT	Accuracy: 96.2%	∙ Synthetic dataset ∙ High computational cost ∙ Communication overhead

The performance metrics reported in the studies summarized in Table 3 were promising. However, caution is warranted when comparing results across studies because of differences in datasets, leukemia subtypes, prediction tasks, sample sizes, and validation methods. These methodological differences can significantly affect reported performance and limit direct comparisons between studies. Despite these differences, the comparison reveals several important trends in the existing literature. A significant portion of the current literature is dedicated to microscopic imaging data, reflecting the availability of annotated blood smear datasets and the high potential of deep learning models to extract morphological features of leukemic cells. Genomic and transcriptomic modalities, in turn, are mainly applied to the analysis of mutation patterns and molecular biomarkers, which allow identifying subtypes and characterizing them at the molecular level. The use of clinical laboratory data, such as Complete Blood Count (CBC) parameters, in machine learning models to predict diagnostic outcomes and assess risk is prevalent because it is widely used in clinical practice. Moreover, flow cytometry-based methods offer high-resolution immunophenotypic data that is necessary to classify precisely, but their application is comparatively low because of standardization issues and data limitations.

Moreover, the research covers various types of diseases, such as Acute Myeloid Leukemia (AML), Acute Lymphoblastic Leukemia (ALL), Chronic Myeloid Leukemia (CML), and Chronic Lymphocytic Leukemia (CLL). It also covers tasks such as cell classification, subtype identification, and prognosis prediction. Overall, these results indicate that although individual modalities provide useful but complementary information, no single source of data can be used to describe the heterogeneity of leukemia. This weakness highlights the growing importance of multimodal artificial intelligence systems that combine diverse biomedical data to produce more precise, generalizable, and clinically resilient diagnostic systems.

### 4.2 Evaluation strategies and clinical alignment

Since the data modalities employed in the diagnosis of leukemia are diverse, suitable evaluation plans are necessary to determine the reliability and clinical usefulness of AI-based diagnostic systems. The most common classification measures used to evaluate the performance of models in leukemia diagnosis are accuracy, precision, recall, F1-score and the Area Under the Curve (AUC). These are used to describe the performance of models to detect leukemic blast cells in microscopic images or to classify leukemia subtypes based on molecular and clinical features. Additionally, cross-validation and test data are often used to evaluate the model’s stability and generalisability. But beyond the algorithm’s performance, clinical relevance is also crucial. The successful deployment of AI systems for leukemia diagnosis should be tested on clinically relevant data, model predictions should be explainable, and the systems should integrate with existing hematological diagnostic workflows. These observations highlight the need for increasingly standardised data, robust validation strategies and models with interpretable clinical implications to ensure the seamless integration of AI systems into routine practice in leukemia diagnosis.

### 4.3 Challenges and future directions

While the artificial intelligence methods for leukemia diagnosis have made significant progress, there are still some problems that limit their clinical applications. As discussed in the previous sections, the current studies are constrained by data availability, multimodal data fusion, generalization and interpretability. These issues must be addressed to develop reliable and clinically applicable AI diagnostic systems.

#### 4.3.1 Dataset availability and standardization

Many current AI models for leukemia diagnosis are trained on relatively small or single-institutional datasets. The staining, imaging, sequencing, and laboratory protocols are highly variable, which can introduce substantial heterogeneity across datasets and affect model performance. Future work should focus on the creation of large, well-annotated, and standardized multi-institutional datasets to enable the development of models and benchmarking.

#### 4.3.2 Multimodal data integration

Leukemia diagnosis in clinical practice is based on the integration of different complementary data, including morphological, immunophenotypic, molecular, and clinical laboratory data. However, most of the current AI studies are based on single-modality data, and multimodal data with imaging, genomic and clinical data of the same patient are scarce. Future AI research should aim to develop multimodal AI systems and multimodal datasets that will enable the integration of multimodal biomedical data.

#### 4.3.3 Generalization and cross-institution validation

The current literature mainly assesses AI models on single-centre data or with limited validation data, which may not represent the variability of clinical practice. Differences in patient demographics, laboratory practices and data collection methods may impact model performance on external data. To establish the validity and robustness of AI-based systems of leukemia diagnosis, future research should focus on multi-centre validation and standardised evaluation procedures.

#### 4.3.4 Model interpretability and clinical trust

Although deep learning models are very effective in diagnosing and classifying leukemia, the decision-making process is often opaque. To facilitate the adoption of these systems in clinical practice, it is necessary to increase their transparency to help clinicians interpret and trust the predictions. Explainable AI methods such as attention visualisation, feature attribution, and saliency mapping can enhance transparency by providing insights into the factors that influence model predictions. Further research should aim to build models that are interpretable and provide explanations of the diagnosis.

#### 4.3.5 Clinical translation and deployment

To successfully integrate AI systems into routine hematological diagnostics, some practical considerations, including regulatory concerns, integration with clinical information systems and user-friendly decision-support interfaces, should be taken into account. To transform existing research progress into clinically valid diagnostic instruments, collaboration among clinicians, biomedical researchers, and AI developers will be necessary.

In addition to the challenges discussed above, there are several emerging learning paradigms that can impact future leukemia diagnostic systems. The great appeal of Self-Supervised Learning (SSL) is that there are large collections of blood smears, bone marrow, genomic, and clinical data that remain only partially annotated. The ability of SSL to learn from unlabelled data can reduce the need for extensive manual labelling while preserving informative representations of features. Another practical benefit of Multiple-Instance Learning (MIL) is that predictions can be made at the sample or slide level, rather than annotating each cell. These methods may be complementary to current CNN, transformer, and graph-based methods and help to make diagnostic workflows more scalable as larger and more diverse leukemia datasets become available.

Recent studies have demonstrated good diagnostic performance, but implementing these models in clinical practice remains a challenge. The majority of published systems have been tested on retrospective data, with limited evidence from prospective clinical studies. Consequently, many proposed models remain research tools rather than technologies routinely used in hospitals. Of the modalities reviewed, image-based modalities, especially those based on peripheral blood smear analysis, seem most promising for clinical implementation, as digital microscopy workflows are already in place in many laboratories. Standardisation of flow cytometry data also supports the practical application of flow cytometry-based approaches. Multimodal systems, however, can face other challenges, such as the complexity of data integration and compatibility with current clinical workflows. There would also be regulatory requirements for AI-based diagnostic software that must be met before it can be deployed in clinical practice, such as the United States Food and Drug Administration (FDA) Software as a Medical Device (SaMD) pathway and the European In Vitro Diagnostic Regulation (IVDR). Future clinical adoption will depend on the model’s performance, external validation, regulatory approval, and integration into routine laboratory use. AI-assisted digital morphology is useful for white blood cell differential analysis, blast identification, and abnormal cell detection on commercially available hematology platforms such as Scopio and Techcyte (68, 69). Their clinical use is successful, underscoring the need for rigorous clinical validation and integration into the clinical laboratory workflow before general clinical use.

AI diagnostic systems for leukemia, besides regulatory clearance and technical performance, need to also account for ethical and operational factors before they can be widely adopted. These models will require prospective clinical validation to ensure their reliability and safety in real-world settings. Issues of responsibility and accountability in the case of diagnostic errors remain relevant, especially when AI systems are employed to assist in clinical decision-making. Furthermore, ethical considerations, including the protection of patient privacy and data security, need to be addressed during the development and deployment of the model. Additionally, the seamless integration of AI into current clinical workflows will be crucial for its successful implementation, ensuring that healthcare professionals can leverage AI without significantly disrupting their routine clinical practice.

### 4.4 Conclusion

Deep learning and artificial intelligence have made a substantial contribution to computational methods of leukemia diagnosis and risk stratification. The review summarized the recent advances in AI-based leukemia diagnostics in various biomedical data modalities, such as microscopic imaging, genomic and transcriptomic profiling, clinical laboratory parameters, and flow cytometry. Deep learning models based on imaging have achieved high levels of automated blast detection and morphological classification, and molecular data analysis has enabled more accurate identification of genetic changes and prognostic biomarkers. Simultaneously, machine learning models applied to clinical and cytometric data have improved subtype classification and diagnostic decision support. Although these developments have been made, most of the current research is confined to unimodal studies, but clinical diagnosis of leukemia necessitates the combination of morphological, immunophenotypic, molecular, and clinical data. The development of multimodal AI models is a promising way to overcome this limitation by allowing the characterization of the disease in a complete manner. Further work on large standardized datasets, multimodal learning systems that are robust and can be interpreted clinically will be critical to the translation of AI-based leukemia diagnostic systems into everyday hematological practice.
