# Supplementary material for: Recent advances in deep learning for leukemia diagnosis: a scoping review of diagnostic modalities and fusion-based approaches
Source: Front Digit Health. 2026 Aug 7;8:1874695. doi: 10.3389/fdgth.2026.1874695 (PMC13494381; doi:10.3389/fdgth.2026.1874695)
Supplement: Supplementary file 1 [file Table1.docx]

**Supplementary Table S1. Detailed database search strategies used in this study.**

| **Database** | **Search Strategy** |
| --- | --- |
| **PubMed** | ("leukemia"[Title/Abstract] OR "acute myeloid leukemia"[Title/Abstract] OR "acute lymphoblastic leukemia"[Title/Abstract])AND("artificial intelligence"[Title/Abstract] OR "machine learning"[Title/Abstract] OR "deep learning"[Title/Abstract] OR "convolutional neural network"[Title/Abstract])AND("blood smear"[Title/Abstract] OR microscopy[Title/Abstract] OR "flow cytometry"[Title/Abstract] OR "RNA-seq"[Title/Abstract] OR "gene expression"[Title/Abstract] OR genomics[Title/Abstract] OR prognosis[Title/Abstract] OR classification[Title/Abstract] OR diagnosis[Title/Abstract] OR "clinical laboratory"[Title/Abstract] OR "multi-omics"[Title/Abstract])  **Filters:** English; Publication date from 2023/01/01 to 2026/02/28 |
| **Scopus** | (TITLE-ABS-KEY(leukemia OR "acute myeloid leukemia" OR "acute lymphoblastic leukemia"))AND(TITLE-ABS-KEY("deep learning" OR "convolutional neural network" OR "machine learning" OR "artificial intelligence"))AND(TITLE-ABS-KEY("blood smear" OR microscopy OR "flow cytometry" OR "RNA-seq" OR "gene expression" OR genomics OR prognosis OR classification OR diagnosis OR "clinical laboratory" OR "multi-omics"))AND (PUBYEAR > 2022)AND (DOCTYPE(ar))AND (LIMIT-TO(LANGUAGE, "English")) |
| **Web of Science** | TS=(leukemia OR "acute myeloid leukemia" OR "acute lymphoblastic leukemia")ANDTS=("deep learning" OR "machine learning" OR "artificial intelligence" OR "convolutional neural network")ANDTS=("blood smear" OR microscopy OR "flow cytometry" OR "RNA-seq" OR "gene expression" OR genomics OR prognosis OR classification OR diagnosis OR "clinical laboratory" OR "multi-omics")  **Refined by:** Document type: Article; Language: English; Timespan: 2023–2026 |
| **IEEE Xplore** | ("All Metadata":"leukemia" OR "acute myeloid leukemia" OR "acute lymphoblastic leukemia")AND("All Metadata":"deep learning" OR "machine learning" OR "artificial intelligence" OR "convolutional neural network")AND("All Metadata":"blood smear" OR microscopy OR "flow cytometry" OR "RNA-seq" OR "gene expression" OR genomics OR prognosis OR classification OR diagnosis OR "clinical laboratory" OR "multi-omics")  **Filters:** Journals; English; 2023–2026 |
